# Hormonal biomarkers for the noninvasive diagnosis of endometriosis: A protocol for a network meta-analysis of diagnostic test accuracy

**DOI:** 10.1097/MD.0000000000012898

**Published:** 2018-10-19

**Authors:** Minghui Shen, Ya Gao, Xueni Ma, Bo Wang, Jiarui Wu, Jiancheng Wang, Jipin Li, Jinhui Tian, Junhai Jia

**Affiliations:** a Department of Clinical Laboratory, Second Hospital of Lanzhou University; b Evidence-Based Medicine Center, School of Basic Medical Sciences, Lanzhou University; c The Second Clinical Medical College of Lanzhou University; d Department of Nursing, Rehabilitation Center Hospital of Gansu Province, Lanzhou; e Department of Clinical Pharmacology of Traditional Chinese Medicine, School of Chinese Materia Medica, Beijing University of Chinese Medicine, Beijing; f Gansu Provincial Hospital; g Emergency Department, Rehabilitation Center Hospital of Gansu Province, Lanzhou, China.

**Keywords:** diagnostic test accuracy, endometriosis, hormonal biomarkers, network meta-analysis

## Abstract

**Background:**

Endometriosis is a major cause of disability and compromised the quality of life in women and teenage girls. The gold standard for diagnosis of endometriosis is laparoscopy with histology of excised endometriosis lesions. However, women can suffer for 8 to 12 years before obtaining a correct diagnosis. Several biomarkers showed good diagnostic value for endometriosis, but no studies directly or indirectly compare the diagnostic value of different biomarkers. We perform this network meta-analysis (NMA) to assess the diagnostic accuracy of hormonal biomarkers, and to find a most effective hormonal biomarker for the diagnosis of endometriosis.

**Methods:**

A systematic search will be performed using PubMed, EMBASE, Cochrane Library and Chinese Biomedicine Literature to identify relevant studies from inception to August 2018. We will include random controlled trials, cross-sectional studies, case-control studies, and cohort studies that evaluated the diagnostic accuracy of hormonal markers for endometriosis. The Quality Assessment of Diagnostic Accuracy Studies 2 quality assessment tool will be used to assess the risk of bias in each study. Standard pairwise meta-analysis and NMA will be performed using STATA V.12.0, MetaDiSc 1.40 and R 3.4.1 software to compare the diagnostic efficacy of different hormonal biomarkers.

**Results:**

The results of this study will be published in a peer-reviewed journal.

**Conclusion:**

This study will summarize the direct and indirect evidence to determine the diagnostic accuracy of the hormonal biomarkers for endometriosis and attempt to find a most effective biomarker for the diagnosis of endometriosis.

**Ethics and dissemination:**

Ethics approval and patient consent are not required as this study is a meta-analysis based on published studies.

**PROSPERO registration number:**

CRD42018105126.

## Abstract 2

Supplemental Digital Content is available in the text

## 1 Introduction

Endometriosis is a common, benign, estrogen-dependent, chronic gynecological disorder associated with pelvic pain and infertility.^[1]^ It is characterized by the presence of endometrial glands and stroma outside of the uterine cavity, primarily on the pelvic peritoneum, ovaries, and rectovaginal septum, and in rare cases on the diaphragm, pleura, and pericardium.^[1–4]^ Endometriosis develops mostly in women of reproductive age and regresses after menopause. Although the exact prevalence is not clear, 5% to 10% of women of reproductive age are estimated to have endometriosis.^[4]^ Based on community prevalence estimates of symptoms,^[5,6]^ endometriosis probably affects 10% of women and 30% to 50% of symptomatic premenopausal women.^[7,8]^ Risk factors for endometriosis include obstruction of menstrual outflow, exposure to diethylstilbestrol in utero, prolonged exposure to endogenous estrogen, short menstrual cycles, low birth weight, and exposure to endocrine-disrupting chemicals.^[9–13]^ Endometriosis is a major cause of disability and compromised quality of life in women and teenage girls.^[14]^ In the United States, the direct costs of endometriosis are estimated at $2801 per woman.^[15]^

Although there is an overall delay of approximately 10 years from symptom onset to diagnosis, endometriosis can be suspected through patient history as well as signs and symptoms.^[1,16]^ The diagnosis may be overlooked in primary care by using this method, which reduces the quality of life of patients.^[8,17]^ At the present moment, the gold standard for diagnosis of endometriosis is laparoscopy with histology of excised endometriosis lesions, and a scoring system has been developed to assess extent of disease.^[1,16]^ However, women can suffer for 8 to 12 years before obtaining a correct diagnosis and treatment.^[18,19]^ Fortunately, in the past few years, researchers have struggled to find a noninvasive way to diagnose endometriosis. Kitawaki et al^[20]^ and Dheenadayalu et al^[21]^ showed that the expression of aromatase in eutopic endometrium as a target for screening test is likely to impair clinical application. Cunha-Filho et al^[22]^ and Lima et al^[23]^ reported that serum prolactin levels are significantly elevated in patients with endometriosis infertility. The study of Cheng et al^[24]^ indicated that CA-125 is an important marker for the diagnosis of endometriosis; however, its sensitivity (SEN) is very low.

Recently, 3 Cochrane Systematic Reviews evaluated the diagnostic value of blood biomarkers, endometrial biomarkers, and combined noninvasive tests for the diagnosis of endometriosis.^[25–27]^ Several biomarkers showed good diagnostic value, but no studies directly or indirectly compare the accuracy of different biomarkers. Therefore, it is not clear which individual biomarker or combined biomarker is most effective for detecting endometriosis. Network meta-analysis (NMA) has the advantage of allowing indirect comparisons of multiple interventions for estimation and ranking their orderings, even though direct head-to-head comparison studies are lacking.^[28]^ We will apply NMA to integrate direct and indirect comparisons,^[29,30]^ which can show the diagnostic accuracy of different index tests clearly.

The objectives of this systematic review and NMA are to determine the diagnostic accuracy of hormonal biomarkers for endometriosis and to compare the diagnostic accuracy of different index tests and to determine which one is the optimal modality for the diagnosis of endometriosis.

## 2 Methods

### 2.1 Design and registration

We will conduct an NMA of diagnostic test accuracy. We registered on the international prospective register of systematic review (PROSPERO)^[31]^ to publish our study protocol. We will follow the Preferred Reporting Items for Systematic Reviews and Meta-analysis statements for reporting our systematic review.^[32]^

### 2.2 Information sources

A systematic search will be performed using PubMed, Embase, Cochrane Library, and Chinese Biomedicine Literature to identify relevant studies from inception to August 2018. There will be no limitations on the year of publication and publication languages. The references of relevant systematic reviews will be searched to identify additional potential studies.

### 2.3 Search strategy

The search terms will include: 17-β hydroxysteroid dehydrogenase, CYP19, aromatase cytochrome P450, estrogen receptor, estrogen sulfotransferase, leucine-rich G protein-coupled receptor 7, relaxin, anti-Mullerian hormone, androgen receptor, progesterone receptor, prolactin, gonadotropin-releasing hormone, chorionic gonadotropin, hormonal marker, SEN, specificity (SPE), false positive (FP) reactions, false negative (FN) reactions, ROC curve, predictive value, endometriosis, adenomyosis, and their synonyms. The search strategy of PubMed can be found in supplementary 1.

### 2.4 Eligibility criteria

#### 2.4.1 Types of studies

We will include random controlled trials, cross-sectional studies, case-control studies, and cohort studies that evaluated the diagnostic accuracy of hormonal markers for endometriosis. These may be either prospective or retrospective. There are no limitations in minimal quality, minimal sample size, or the number of patients.

#### 2.4.2 Type of patients

Study participants will include women with suspected endometriosis based on clinical symptoms, pelvic examination, or both, who undertook the index test as well as the reference standard. All participants receive one or several index tests. There are no limitations in age, race, or nationality.

#### 2.4.3 Type of index tests

Any type of hormonal biomarker aimed at evaluating the diagnostic value for endometriosis. The index test can be one hormonal biomarker or 1 hormonal biomarker combines with other tests.

#### 2.4.4 Reference standards

The reference standard is the visualization of endometriosis at surgery (laparoscopy or laparotomy) with or without histological confirmation, because this is currently the best available test for endometriosis.

#### 2.4.5 Type of outcomes

The primary outcomes are SEN, SPE, positive predictive value, negative predictive value, positive likelihood ratio (PLR), negative likelihood ratio (NLR), diagnostic odds ratio (DOR), area under the curve, and their respective 95% confidence intervals. The second outcomes are relative diagnostic estimates of different hormonal biomarkers.

#### 2.4.6 Other criteria

There will be no limitations on language, publication year, and publication status.

### 2.5 Study selections

We will import the literature search records into ENDNOTE X7 literature management software. Two independent reviewers will examine the title and abstract of studies found in the search to identify related studies. Then, the same 2 reviewers will retrieve the full text of all possibly relevant studies and assess the eligibility of each study according to the eligibility criteria. Conflicts will be resolved by a third reviewer.

### 2.6 Data items

A draft data extraction sheet will be developed using Microsoft Excel 2013 (Microsoft Corp, Redmond, WA, www.microsoft.com). Two trained reviewers will independently extract data from the included studies, and another trained reviewer will check the extracted data. We will resolve discordant evaluations by discussion to reach consensus. Data will be extracted from eligible studies including general information such as author name, year of publication, country of the first author, number of authors, journal name, country of journal, funding, and types of studies; characteristics of study including age and number of participants, number and name of index test, and number and name of reference test; and the reported number of TPs, FNs, TNs, and FPs. If studies did not report these values, we will attempt to reconstruct the 2 × 2 tables from the diagnostic estimates presented in the article for each index test.

### 2.7 Assessment of risk of bias in included studies

The Quality Assessment of Diagnostic Accuracy Studies 2 quality assessment tool will be used to assess the methodological quality.^[33]^ Two review authors will independently assess the risk of bias in each study according to predefined criteria. We will resolve any disagreement by discussion or by involving a third assessor.

### 2.8 Geometry of the network

A network plot will be drawn to describe and present the geometry of index tests using R software V.3.4.1. Trials will be excluded if they are not connected by index tests. Nodes in network geometry represent different hormonal biomarkers and edges represent head to head comparisons. The size of nodes and thickness of edges are associated with sample sizes of index tests and numbers of included trials, respectively.

### 2.9 Network meta-analysis

#### 2.9.1 Pairwise meta-analyses

We will construct forest plots showing estimates of SEN, SPE (SPE), PLR, NLR, DOR, and their corresponding 95% confidence intervals for each index tests using STATA V.12.0 (Stata) and MetaDiSc 1.40. The heterogeneity between each study will be estimated using the Q value and the inconsistency index (*I*^2^ test). If the *I*^2^ is ≤50%, it suggests that there is negligible statistical heterogeneity, and the fixed effects model will be employed. If the *I*^2^ is >50%, we will explore sources of heterogeneity by subgroup analysis and meta-regression. If there is no clinical heterogeneity, the random effects model will be used to perform the meta-analysis. We will also plot sensitivities and specificities in the summary receiver operating characteristic (SROC) space, using different symbols for different hormonal biomarkers. In addition, we will use STATA V.12.0 (Stata) and Review Manager 5.30 (RevMan) analysis software to build the hierarchical SROCs graphics for each index test.

#### 2.9.2 Indirect comparisons between competing diagnostic tests

We will calculate relative diagnostic outcomes between index tests including relative SEN, relative SPE, relative DOR, relative PLR, and relative NLR. Then, we will conduct indirect comparisons using the relative diagnostic outcomes. All analysis will be performed using STATA V.12.0 (Stata) software.

#### 2.9.3 Assessment of publication bias

The effective sample size funnel plot and associated regression test of asymmetry will be conducted to detect publication bias where there are >10 studies available for an index test.^[34]^

#### 2.9.4 Subgroup analysis

If sufficient studies are available, we will perform meta-regression or subgroup analysis on the basis of the age, body mass index, and ethnicity of participants; the country in which the study was conducted; the time period of index tests; and the risk factors of endometriosis.

## 3 Discussion

A diagnostic test without the need for surgery will reduce the associated surgical risks, increase accessibility to a diagnostic test and improve treatment outcomes.^[25]^ Although multiple markers and imaging techniques have been explored as diagnostic tests for endometriosis, none of them have been implemented routinely in clinical practice and many have not been subject to systematic review.^[27]^ This NMA will summarize the direct and indirect evidence to assess the diagnostic accuracy of the hormonal biomarkers for endometriosis and attempt to find a most effective biomarker for the diagnosis of endometriosis. We hope that these tests can replace diagnostic surgery and help clinicians make more accurate diagnostic decisions.

## Author contributions

MS, YG, JT, and JJ planed and designed the research. XM, BW, JW, JW, and JL tested the feasibility of the study. MS, YG, and JJ wrote the manuscript. All authors approved the final version of the manuscript.

**Conceptualization:** Minghui Shen.

**Investigation:** Minghui Shen.

**Methodology:** Ya Gao, Xueni Ma, Jiancheng Wang, Jipin Li, Jinhui Tian, Junhai Jia.

**Project administration:** Junhai Jia.

**Resources:** Ya Gao, Xueni Ma, Jiarui Wu.

**Software:** Bo Wang.

**Supervision:** Jipin Li, Junhai Jia.

**Validation:** Jiarui Wu, Jiancheng Wang.

**Visualization:** Bo Wang.

**Writing – original draft:** Minghui Shen, Ya Gao.

**Writing – review and editing:** Jinhui Tian, Junhai Jia.

## Supplementary Material

Supplemental Digital Content
