# Supplementary material for: Hormonal biomarkers for the noninvasive diagnosis of endometriosis: A protocol for a network meta-analysis of diagnostic test accuracy
Source: Medicine (Baltimore). 2018 Oct 19;97(42):e12898. doi: 10.1097/MD.0000000000012898 (PMC6211906; doi:10.1097/MD.0000000000012898)
Supplement: Supplemental Digital Content [file medi-97-e12898-s001.docx]

**Hormonal biomarkers for the non-invasive diagnosis of** **endometriosis: A protocol for a network meta-analysis of diagnostic test accuracy**

Minghui Shen, MD^a^, Ya Gao, MM^b^, Xueni Ma, MD^c^, Bo Wang, MD^d^, Jiarui Wu, MD^e^, Jiancheng Wang, MD^f^, Jipin Li, MD^c^, Jinhui Tian, PhD^b^, Junhai Jia, MD^g,*^

**Search strategy**

#1 "17β HSD"[Title/Abstract] OR "17-β hydroxysteroid dehydrogenase"[Title/Abstract]

#2 "17β HSD2"[Title/Abstract] OR "17-β hydroxysteroid dehydrogenase type 2"[Title/Abstract]

#3 "Cytochrome P450 Family 19"[Mesh] OR "CYP19"[Title/Abstract] OR "aromatase cytochrome P450"[Title/Abstract]

#4"Receptors, Estrogen"[Mesh] OR ER[Title/Abstract] OR "estrogen receptor"[Title/Abstract] OR "oestrogen receptor"[Title/Abstract] OR "estrogen receptors"[Title/Abstract] OR "oestrogen receptors"[Title/Abstract]

#5 "Estrogen Receptor alpha"[Mesh] OR ER-α[Title/Abstract] OR "oestrogen receptor-alpha"[Title/Abstract] OR "estrogen receptor-alpha"[Title/Abstract] OR "oestrogen receptor alpha"[Title/Abstract] OR "estrogen receptor alpha"[Title/Abstract]

#6 "Estrogen Receptor beta"[Mesh] OR ER-β[Title/Abstract] OR "oestrogen receptor-beta"[Title/Abstract] OR "estrogen receptor-beta"[Title/Abstract] OR "oestrogen receptor beta"[Title/Abstract] OR "estrogen receptor beta"[Title/Abstract]

#7 EST[Title/Abstract] OR "oestrogen sulphotransferase"[Title/Abstract] OR "estrogen sulphotransferase"[Title/Abstract] OR "oestrogen sulfotransferase"[Title/Abstract] OR "estrogen sulfotransferase"[Title/Abstract]

#8 LGR7 [Title/Abstract] OR "leucine-rich G protein-coupled receptor 7"[Title/Abstract]

#9 "Relaxin"[Mesh] OR Relaxin[Title/Abstract]

#10"Anti-Mullerian Hormone"[Mesh] OR AMH[Title/Abstract] OR "anti-mullerian hormone"[Title/Abstract]

#11"Receptors, Androgen"[Mesh] OR "AR"[Title/Abstract] OR "androgen receptor"[Title/Abstract] OR "androgen receptors"[Title/Abstract]

#12"Receptors, Progesterone"[Mesh] OR PR[Title/Abstract] OR "progesterone receptor"[Title/Abstract] OR "progesterone receptors"[Title/Abstract] OR "progestogen receptor"[Title/Abstract]

#13"Prolactin"[Mesh] OR PRL[Title/Abstract] OR prolactin[Title/Abstract] OR lactogen[Title/Abstract]

#14"Gonadotropin-Releasing Hormone"[Mesh] OR GnRH[Title/Abstract] OR "Gonadotropin releasing hormone"[Title/Abstract] OR "Gonadotropin-releasing hormone"[Title/Abstract]

#15"Chorionic Gonadotropin"[Mesh] OR "HCG-beta, des-(122-145)-" [Supplementary Concept] OR "chorionic gonadotrophin"[Title/Abstract] OR HCG[Title/Abstract]

#16 "hormonal marker"[Title/Abstract] OR "hormonal markers"[Title/Abstract]

#17 #1 OR #2 OR #3 OR #4 OR #5 OR #6 OR #7 OR #8 OR #9 OR #10 OR #11 OR #12 OR #13 OR #14 OR #15 OR #16

#18 "Sensitivity AND Specificity"[Mesh] OR "False Positive Reactions"[Mesh] OR "False Negative Reactions"[Mesh] OR "ROC Curve"[Mesh] OR "Predictive Value of Tests"[Mesh] OR sensitivity[Title/Abstract] OR specificity[Title/Abstract] OR receiver operating characteristic[Title/Abstract] OR receiver operator characteristic[Title/Abstract] OR predictive value*[Title/Abstract] OR roc[Title/Abstract] OR pre-test odds[Title/Abstract] OR pretest odds[Title/Abstract] OR pre-test probability*[Title/Abstract] OR pretest probability*[Title/Abstract] OR post-test odds[Title/Abstract] OR posttest odds[Title/Abstract] OR post-test probabilit*[Title/Abstract] OR posttest probabilit*[Title/Abstract] OR likelihood ratio*[Title/Abstract] OR positive predictive value*[Title/Abstract] OR negative predictive value*[Title/Abstract] OR false negative*[Title/Abstract] OR false positive*[Title/Abstract] OR true negative*[Title/Abstract] OR true positive*[Title/Abstract] OR fn[Title/Abstract] OR

fp[Title/Abstract] OR tn[Title/Abstract] OR tp[Title/Abstract]

#19 "Endometriosis"[Mesh] OR Endometrio*[Title/Abstract]

#20 "Adenomyosis"[Mesh] OR "adenomyosis"[Title/Abstract]

#21 #19 OR #20

#22 #17 AND #18 AND #21
